# Improving Ponzi Scheme Contract Detection Using Multi-Channel TextCNN and Transformer

**DOI:** 10.3390/s21196417

**Published:** 2021-09-26

**Authors:** Yizhou Chen, Heng Dai, Xiao Yu, Wenhua Hu, Zhiwen Xie, Cheng Tan

**Affiliations:** 1 School of Computer Science and Artificial Intelligence, Wuhan University of Technology, Wuhan 430070, China; chenyizhou_whu_cs@163.com (Y.C.); cheng_tan@whut.edu.cn (C.T.); 2 School of Computer Science, Wuhan University, Wuhan 430072, China; xiezhiwen@whu.edu.cn; 3 School of Mechanical and Electrical Engineering, Wuhan Qingchuan University, Wuhan 430204, China; daiheng726@163.com; 4 Wuhan University of Technology Chongqing Institute, Chongqing 401120, China

**Keywords:** blockchain, smart contracts, Ponzi schemes, structured sequences, deep learning

## Abstract

With the development of blockchain technologies, many Ponzi schemes disguise themselves under the veil of smart contracts. The Ponzi scheme contracts cause serious financial losses, which has a bad effect on the blockchain. Existing Ponzi scheme contract detection studies have mainly focused on extracting hand-crafted features and training a machine learning classifier to detect Ponzi scheme contracts. However, the hand-crafted features cannot capture the structural and semantic feature of the source code. Therefore, in this study, we propose a Ponzi scheme contract detection method called MTCformer (Multi-channel Text Convolutional Neural Networks and Transofrmer). In order to reserve the structural information of the source code, the MTCformer first converts the Abstract Syntax Tree (AST) of the smart contract code to the specially formatted code token sequence via the Structure-Based Traversal (SBT) method. Then, the MTCformer uses multi-channel TextCNN (Text Convolutional Neural Networks) to learn local structural and semantic features from the code token sequence. Next, the MTCformer employs the Transformer to capture the long-range dependencies of code tokens. Finally, a fully connected neural network with a cost-sensitive loss function in the MTCformer is used for classification. The experimental results show that the MTCformer is superior to the state-of-the-art methods and its variants in Ponzi scheme contract detection.

## 1. Introduction

Blockchain is an emerging technology that plays an important role in decentralized technologies and applications, such as storage, calculation, security, interaction, and transactions. Since 2008, along with the increasing popularity of cryptocurrencies (e.g., Bitcoin and Ether) in the financial market, the related blockchain technology has also been maturing and developing and has become one of the most promising network information technologies for ensuring security and privacy [1,2]. As opposed to conventional security schemes that focus on the path traversed by data, the blockchain is essentially a decentralized shared ledger, which focuses on protecting data and providing immutability and authentication.

Smart contracts, as the programs running on the blockchain, have been applied in a variety of business areas to achieve automatic point-to-point trustable transactions [3,4,5].

A number of blockchain platforms, such as Ethereum, provide some application program interfaces (APIs) for the development of smart contracts. When a developer deploys a smart contract to blockchain platforms, the source code of the smart contract will be compiled into bytecode and reside on the blockchain platforms [3,4,6,7]. Then, every node on the blockchain can receive the bytecode of the smart contract, and everyone can call the smart contract by sending the transaction to the corresponding smart contract address.

Blockchain and smart contracts have been applied to a variety of fields, such as the Internet of Things (IoT). For example, the Commonwealth Bank of Australia, Wells Fargo, and Brighann Cotton conducted the first interbank trade transaction in the world, which combined IoT, blockchain technologies, and smart contracts (https://www.gtreview.com/news/global/landmark-transaction-merges-blockchain-smart-contracts-and-iot/ accessed on 31 July 2021). They employed IoT technologies with a GPS device to track the geographic location of goods in transit. When the goods reached their final destination, the release of funds was automatically triggered by the smart contracts. With the help of smart contracts, paperwork that takes a few days using manual processes can be completed in minutes, which largely reduces the time cost and improves the trade efficiency. In addition, smart contracts make transactions more transparent, because the transaction data are updated in real time in the same system. Meanwhile, smart contracts cannot be tampered after deployment; thus, security is greatly enhanced, and the risk of fraud is reduced.

However, due to the high complexity of blockchain-related technologies, it is generally difficult for investors to understand the business logic of smart contracts in depth, and they can only comprehend the operation mechanism of the business through some descriptive information about smart contracts. As a result, some speculators have introduced the classic form of financial investment fraud—the Ponzi scheme—into blockchain transactions and brought extremely costly losses to investors. The highly complex smart contract program makes Ponzi schemes more confusing. For the endless stream of smart contract-based Ponzi schemes, a post on a popular Bitcoin forum (bitcointalk.org) showed that more than 1800 Ponzi scheme contracts emerged between June 2011 and November 2016, where the financial losses caused were even harder to estimate [8]. As Ponzi schemes become more prevalent in blockchain transactions, researchers need to find a way to automatically detect Ponzi scheme contracts.

Some existing works in the literature [7,9] focused on manually extracting features from the smart contract code and the transaction history of smart contracts. Specifically, Chen et al. [7,9] compiled the smart contract code to generate bytecode and then decompiled it into operating code (Opcode) using external tools to extract the Opcode features. In addition, they extracted the statistical account features from the transaction history of smart contracts. Finally, the random forest algorithm was used as the classification model based on the composited features to detect Ponzi scheme contracts. However, the source code of smart contracts has a well-defined structure and semantic information, which the hand-crafted features cannot capture well. Therefore, the detection performance of the existing works is not satisfactory enough.

With the development of deep learning technologies, many researchers have tried to apply deep learning algorithms to extract more powerful features from source code to conduct the related tasks. However, to the best of our knowledge, there is no investigation on the significance of deep learning for Ponzi scheme contract detection. The challenges of automatic Ponzi scheme contract detection using deep learning usually include:

**(1) How to extract the structural features of the smart contract code well.**

Using the plain source code of smart contracts as the input ignores the structural information of smart contracts. How to achieve the serialization transformation of the code without destroying the structural semantics of the code and conform to the input requirements of the deep learning model after the transformation is a problem that needs to be considered.

**(2) How to capture the long-range dependencies between code tokens of smart contracts.**

The source code of smart contract in our experimental dataset is very long. For the long sequence training, traditional deep learning models (e.g., LSTM [10] and GRU [11]) have the problem of gradient disappearance. The more relevant the output of the last time step is, the later the input is. Furthermore, an earlier input causes more information to be lost in the transmission process. Obviously, such logic does not make sense in the context of semantic understanding. This phenomenon is manifested in the model as gradient disappearance. Therefore, in the long sequence training, we need a model that can capture the long-range dependencies efficiently and without gradient disappearance.

To address these problems, we propose a Ponzi scheme contract detection approach called MTCformer based on the multi-channel TextCNN (MTC) and Transformer. The MTCformer first parses the smart contract code into an Abstract Syntax Tree (AST). Then, in order to reserve the structural information, the MTCformer employs the Structure-Based Traversal (SBT) method proposed by Hu et al. [12] to convert the AST to the SBT sequence. After that, the MTCformer employs the multi-channel TextCNN to learn feature representations based on neighboring words (tokens) to obtain local structural and semantic feature of the source code. The multi-channel TextCNN contains multiple filters of different sizes, which can learn multiple different dimensions of information and capture more complete local features in the same window. Next, the MTCformer uses Transformer to capture the long-range dependencies between code tokens. Finally, a fully connected neural network with a cost-sensitive loss function is used for classification.

We conduct experiments on a Ponzi scheme contract detection dataset, which contains 200 Ponzi scheme contracts and 3588 non-Ponzi scheme contracts. We extensively compare the performance of the MTCformer against the three recently proposed methods (i.e., Account, Opcode, Account + Opcode). The experimental results show that (1) the MTCformer outperforms Account by 51.56% in terms of precision, 315% in terms of recall, and 297% in terms of F-score; (2) the MTCformer performs better than Opcode by 3.19%, 13.7%, and 8.54% in terms of the three metrics; and (3) the MTCformer also outperforms Account + Opcode by 2.1%, 20.29%, and 12.66% in terms of precision, recall, and F-score, respectively. We also evaluate the MTCformer against the variants, and the experimental results indicate that the MTCformer outperforms its variants in terms of the three metrics.

In summary, the primary contributions of this paper are as follows:

(1) We propose an MTCformer method combining the multi-channel TextCNN and Transformer for Ponzi scheme contract detection. The MTCformer can both extract the local structural and semantic features and capture the long-range dependencies between code tokens.

(2) We compare the MTCformer with the state-of-the-art methods and their variants. The experimental results show that the MTCformer achieves more encouraging results than the compared methods.

The remainder of this paper is organized as follows. Section 2 introduces the related work and background. Section 3 proposes our MTCformer method to detect Ponzi scheme contracts. Section 4 presents the experimental setup and results. Section 5 discusses the impact of the parameters. Finally, Section 6 concludes the paper and enumerates ideas for future studies.

## 2. Related Work and Background

This section provides background information on topics relevant to this paper. Section 2.1 describes the application of blockchain in the Internet of Things (IoTs). Section 2.2 briefly explains the basic concepts of Ethereum and smart contracts. Section 2.3 introduces the related work of the Ponzi scheme contract detection. Section 2.4 briefly introduces the Abstract Syntax Tree and Structure-Based Traversal used to structure the source code of the smart contract. Section 2.6 briefly introduces the text-based Convolutional Neural Network and Transformer.

### 2.1. Blockchain and IoT

Blockchain refers to a series of decentralized and tamper-proof ledgers combined into a network. It provides a service to the end-users with lower transaction costs and without unnecessary intervention. Because of its uniqueness, blockchain has offered many benefits to business and management, such as decentralization, intractability and strategic applications, security and behavior, and operations and strategic decision making [13]. Currently, blockchain is already widely used in the Internet of Things (IoT). Singh et al. [14] used blockchain and artificial intelligence to design and develop IoT architectures to support effective big data analysis. Tsang et al. [15] explored the intellectual cores of the blockchain–Internet of Things (BIoT). Zhang et al. [16] proposed an e-commerce model for IoT E-business to realize the transaction of smart property. Puri et al. [17] designed a strategy based on smart contracts to handle the security and privacy issues in an IoT network. Zhang et al. [18] studied key access control issues in IoT and proposed a smart contract-based model to enable reliable access control for IoT systems. By integrating the IoT with blockchain systems and smart contracts, Ellul et al. [19] provided the automatic verification of physical processes involving different parties.

### 2.2. Ethereum and Smart Contracts

Ethereum is a blockchain platform that provides a Turing-complete programming language (Solidity) and a corresponding runtime environment (i.e., EVM) [20]. The platform allows users to develop blockchain applications using short code [21]. Currently, Ethereum is the largest platform that provides an execution environment for smart contracts [22]. The smart contracts running on Ethereum are a series of EVM bytecodes residing on the blockchain that can be triggered for execution. These bytecodes are compiled by the EVM compiler from the smart contract source code. Deployment is accomplished by uploading bytecode to the blockchain through an Ethereum client. These codes implement certain predefined rules and are “autonomous agents” that exist in the Ethereum execution environment. Once deployed, smart contracts cannot be changed, and the execution of their coding functions produces the same result for anyone running them.

In Ethereum, two types of accounts exist. One is an externally owned account (EOA) and the other is a contract account [23,24]. EOAs have a private key that provides access to the corresponding Ethereum or contract. On the other hand, contract accounts have smart contract codes. Contract accounts cannot run their own smart contracts. Running a smart contract requires an external account to initiate a transaction to the contract account, which initiates the execution of the code within it.

### 2.3. Ponzi Scheme Contract

A Ponzi scheme is a type of investment scam in the financial market. Organizers of Ponzi schemes use the funds of new investors to pay interest and short-term returns to previous investors. The organizers often package the investment project with the illusion of low risk and high and stable returns, which are used to confuse investors who are unfamiliar with the industry or have a fluke mentality.

In the blockchain era, many Ponzi schemes are disguised in smart contracts. We refer to these Ponzi schemes as smart Ponzi schemes and refer to the corresponding smart contracts as Ponzi scheme contracts [9]. Due to their self-executing and non-tamper-evident characteristics, smart contracts have become a powerful tool for Ponzi schemes to attract victims. More importantly, the originators of Ponzi schemes are anonymous.

Machine learning and data mining technologies have been used to detect Ponzi scheme contracts. Ngai et al. [25] proposed a technology based on data mining to detect financial fraud, and it is used for detecting Bitcoin Ponzi schemes [26]. Chen et al. [7,9] used the transaction history of smart contracts in Ethereum and the Opcode of smart contracts as hand-crafted features to detect smart Ponzi schemes. Different from their studies, our paper focuses on automatically learning the hidden rich semantic features from the source code to detect Ponzi scheme contracts by using deep learning and natural language processing technologies.

### 2.4. Abstract Syntax Tree and Structure-Based Traversal

In the field of natural language processing, the processing of text data includes syntactic analysis, lexical analysis, dependency analysis, and machine translation. Generally, ordinary text is unstructured data, which requires to be structured before analysis and understanding. The structured data is more conducive to learning semantic features and dependencies in the text.

An abstract syntax tree (AST) is a tree-like representation of the abstract syntactic structure of the source code, where each node is a construct occurring in the code [27,28,29]. The reason for the abstraction is that the abstract syntax tree does not represent every detail of the appearance of the real syntax. For example, nested brackets are implied in the structure of the tree and are not presented in the form of nodes. In short, it is the conversion of unstructured code into a tree structure according to certain rules.

The Structure-Based Traversal (SBT) method proposed by Hu et al. [12] converts the abstract syntax trees into specially formatted sequences via globally traversing the trees. Existing code representation works [30,31,32] have proved that the SBT method has a strong ability in preserving the code structure and lexical information. Therefore, we also employ the SBT method to structure the source code.

### 2.5. Text-Based Convolutional Neural Network

Convolutional Neural Networks (CNN) were initially applied in the field of computer vision. Subsequently, they have been proven to achieve excellent results in traditional natural language processing field, such as semantic analysis [33,34,35,36], search query [37], sentence modeling [38], etc. The TextCNN is a deep learning algorithm with high performance in feature learning [39].

The core goal of the TextCNN is to capture local features. All words need to be converted to low-dimensional dense vectors. During the training process, if these word vectors are fixed, it is called CNN-static. Otherwise, as the word embeddings are updated, the corresponding model is called CNN-non-static [40]. In general, the *i*-th word can be represented as a *k*-dimensional word vector $x_{i}\in R^{k}$ in the sentence. A sentence of length *n* is expressed as $x_{1:n}={\left[x_{1}^{T},x_{2}^{T},\ldots \;,x_{n}^{T}\right]}^{T}$. In this way, $x_{1:n}$ is similar to an image that can be used as input to CNN. In the convolution layer, many filters with different window sizes are sliding over $x_{i}$. Each filter convolves $x_{1:n}$ to generate a different feature mapping. Correspondingly, for the text, local features are sliding windows consisting of several words, similar to N-grams. The advantage of Convolutional Neural Networks is that they can automatically combine and filter N-gram features to obtain local semantic information at different levels of abstraction [41,42,43,44,45,46]. Then, the maximum pooling operation is applied to the feature mapping to obtain the maximum value as input to the Transformer layer. Generally, some regularization techniques such as dropout and batch normalization can be used after the pooling layer to prevent model overfitting.

### 2.6. Transformer-Related Structures

With traditional RNN-based models (e.g., LSTM, GRU, etc.), the computation can only be done sequentially from left to right or from right to left when text is used as input. There are two problems with this mechanism:

- The computation of time step *t* relies on the results of the computation at moment t−1. This limits the parallel computing capability of the model.
- LSTM and GRU can solve the problem of back-and-forth dependence of long sequences to some extent, but the performance will drop sharply when encountering particularly long sequences.

Both problems are addressed to some extent by the Transformer model [47] proposed by Google in 2017. Unlike CNN and RNN, the entire network structure of the Transformer is composed entirely of the attention mechanism. More precisely, the Transformer only consists of self-attention and a Feed Forward Neural Network. A trainable neural network based on Transformer can be built by stacking the Transformer.

The Transformer model does not need to process words sequentially in sequence and can train all words at the same time, which greatly improves the degree of parallelism and increases the computational efficiency. Furthermore, the attention mechanism pays attention to all words of the whole input sequence, making the model associate the words of the context. It helps the model to encode the text better. However, the attention mechanism itself cannot capture positional information. Therefore, the “positional encoding” approach is proposed. Specifically, positional encoding adds the positional information of words to the word vector and uses the word embedding and positional embedding together as the input of Transformer. It makes the model understand the position of each word in the sentence, not just the semantics of the word itself.

In this paper, Ponzi scheme contract detection is a classification task that does not require the use of the full Transformer model but instead uses positional encoding and an attention encoder for feature learning.

## 3. Smart Ponzi Scheme Detection Model

### 3.1. Overall Process

**Figure 1:**
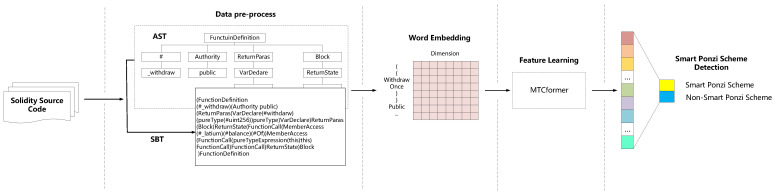
The overall workflow of our proposed MTCformer.

**Figure 2:**
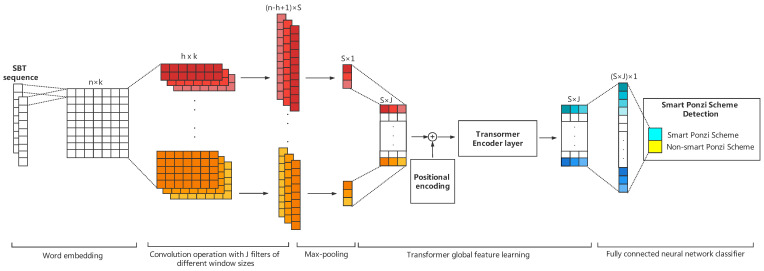
The feature learning process of the MTCformer.

As shown in Figure 1, the overall process of Ponzi scheme contract detection consists of four steps:

- Data pre-processing: The source code of the smart contract is firstly parsed into an Abstract Syntax Tree (AST) according to the ANTLR syntax rules. Then, we employ the SBT method to convert the AST into a SBT sequence to reserve the structure information.
- Word embedding: The pre-processed SBT sequences are fed into the embedding layer for word embedding, and the words (tokens) in each sequence will be converted into fixed dimensional word vectors. Then, an SBT sequence will be converted into word embedding matrices.
- Feature learning: We use the multi-channel TextCNN and Transformer to automatically generate structural and semantic features of smart contract code from the input word embedding matrices. The feature learning process is shown in Figure 2.
- Ponzi scheme contract detection: We use a fully connected layer neural network to do the final classification and conduct a calculation on the real label (presence of a smart Ponzi scheme) to optimize the loss function.

### 3.2. Data Pre-Processing

The source code of the smart contract is in unstructured form; thus, we need to learn the structure features of the smart contract code for better Ponzi scheme contract detection [48,49,50]. Therefore, instead of using the plain source code directly as the input of the model, we parse the source code into an Abstract Syntax Tree (AST) according to the ANTLR [51] syntax rules and then generate a Structure-Based Traversal (SBT) sequence from the AST using the SBT method [12].

The detailed process of the SBT method is as follows:

- Starting with the root node, the method firstly uses a pair of parentheses to represent the tree structure and places the root node itself after the right parenthesis.
- Next, the method traverses the subtree of the root node and places all root nodes of the subtree in parentheses.
- Finally, the method recursively traverses each subtree until all nodes are traversed to obtain the final sequence.

**Figure 3:**
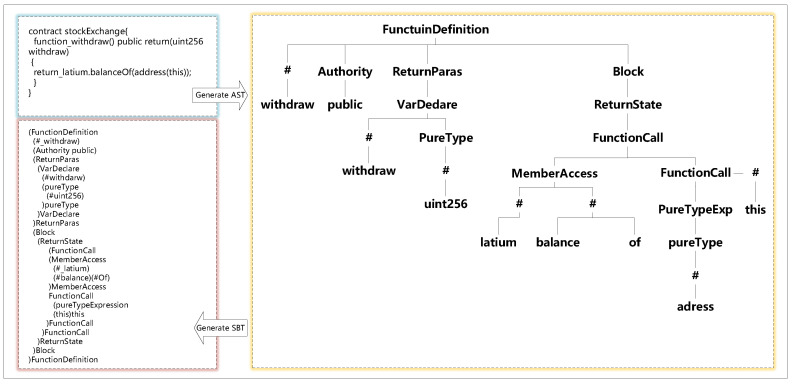
The SBT process.

As shown in Figure 3, we firstly use the parsing tool solidity-parser-antlr (https://github.com/federicobond/solidity-parser-antlr accessed on 31 July 2021) to parse the source code to the AST and then convert the AST to the SBT sequence. The StockExchange contract defines a function called ‘withdraw’, in which non-leaf nodes are represented by type (e.g., the root node of the contract is FunctionDefinition, and variable, function name, return value name, etc., are represented by “#”). The leaf nodes represent the value of each type.

### 3.3. Embedding Layer

The word embedding matrix can be initialized using random initialization or using pre-trained vectors learned by models such as CodeBert [52], Word2Vec [53], GloVe [54], FastText [55], ELMo [56], etc. The pre-trained word embedding can leverage other corpora to obtain more prior knowledge, while word vectors trained by the current network can better capture the features associated with the current task. Random initialization is used in this paper due to the absence of the pre-trained model of the smart contract code.

The $e_{i}\in R^{k}$ is the *k*-dimensional word vector corresponding to the *i*-th word in the SBT sentence. A sequence of length *n* can be expressed as a matrix $E_{1:n}={\left(e_{1}^{T},e_{2}^{T},\ldots \;,e_{n}^{T}\right)}^{T}\in R^{n\times k}$. Then, matrix $E_{1:n}$ is taken as the input to the convolution layer.

### 3.4. Convolutional Layer

In the convolution layer, in order to extract local features, *J* filters of different sizes are convolved on $E_{1:n}$. The width of each filter window is the same as $E_{1:n}$; only the height is different. In this way, different filters can obtain the relationship of words in different ranges. Each filter has S (s∈S) convolution kernels. The Convolutional Neural Networks learn parameters in the convolutional kernel, and each filter has its own focus, so that multiple filters can learn multiple different pieces of information. Multiple convolutional kernels of the same size are designed to learn features that are complementary to each other from the same window. The detailed formula is as follows:

(1) $$C_{i}^{j}=f\left(W^{j}\cdot E_{i:i+h-1}+b\right),$$

where the $W^{j}\in R^{h\times k}$ denotes the weight of the *j*-th (j∈J) filter of the convolution operation, the $C_{i}^{j}$ is the new feature resulting from the convolution operation, b∈R is a bias, and *f* is a non-linear function. Many filters with varying window sizes slide over the full rows of $E_{1:n}$, generating a feature map $\left[C_{1}^{j},C_{2}^{j},\ldots \;,C_{n-h+1}^{j}\right]$. The most important feature value ${\hat{C}}_{s}^{j}$ was obtained by 1-max pooling for one scalar and mathematically written as:

(2) $${\hat{C}}_{s}^{j}=Max\left(\left[C_{1}^{j},C_{2}^{j},\ldots \;,C_{n-h+1}^{j}\right]\right).$$

*S* convolution kernels are computed to obtain *S* feature values, which are concatenated to obtain a feature vector $P^{j}$:

(3) $$P^{j}=\left[{\hat{C}}_{1}^{j},{\hat{C}}_{2}^{j},\ldots \;,{\hat{C}}_{S}^{j}\right].$$

Finally, the feature vector of all filters is stacked into a complete feature mapping matrix $M\in R^{J\times S}$:

(4) $$M=[P^{1},P^{2},\ldots \;,P^{j}],$$

which is used as the input of the Transformer layer. Generally, some regularization techniques such as dropout and batch normalization can be imposed after the pooling layer to prevent model overfitting [40].

### 3.5. Transformer

Since the multi-head attention is not the convolution and recurrent structure, it needs position encoding to utilize the sequence order of the feature matrix *M*. This kind of positional encoding rule is as follows:

(5) $$PE_{\left(pos,2i\right)}=sin\left(\frac{pos}{{10,000}^{\frac{2i}{d}}}\right),$$

(6) $$PE_{\left(pos,2i+1\right)}=cos\left(\frac{pos}{{10,000}^{\frac{2i+1}{d}}}\right),$$

where pos is the token position in the sequence, *i* is the dimension index, *d* is the dimensions of the complete feature mapping *M*, and PE is the position encoding matrix isomorphic to *M*.

The matrix PE+M is fed into multi-head attention to capture long-range dependencies. The details are given by the following equations:

(7) $$q_{1},q_{2},\ldots \;q_{J}=split\left(QW^{Q}\right)$$

(8) $$k_{1},k_{2},\ldots \;k_{J}=split\left(KW^{K}\right)$$

(9) $$v_{1},v_{2},\ldots \;v_{J}=split\left(VW^{V}\right)$$

(10) $$head_{j}=Softmax\left(\frac{q_{j}k_{j}^{T}}{\sqrt{d_{K}}}\right)$$

(11) $$MultiHead\left(Q,K,V\right)=Concat\left(head_{1},head_{2},\ldots \;,head_{J}\right)W^{o}$$

The matrices of queries, keys, and values are denoted by $Q\in R^{Q_{l}\times Q_{d}},\;K\in R^{K_{l}\times K_{d}},\;V\in R^{V_{l}\times V_{d}}$, respectively, while $q_{j}\in R^{Q_{l}\times q_{d}},\;k_{j}\in R^{K_{l}\times k_{d}},\;v_{j}\in R^{V_{l}\times v_{d}}$ represent their splitted matrices for $head_{j}$. Specifically, $q_{d}=k_{d}=v_{d}=\frac{d_{model}}{J}$. The $W^{Q}\in R^{Q_{d}\times d_{model}},\;W^{K}\in R^{K_{d}\times d_{model}},\;W^{V}\in R^{V_{d}\times d_{model}}$ are three weight trainable matrices. Equation (10) describes the output of $head_{j}$. After the concatenating from all heads and the linear transformation with $W^{o}\in R^{J_{v_{d}}\times d_{model}}$, we can obtain the output of the multi-head attention [47,57].

Then, the output of the multi-head attention is delivered to the FFN (Feed Forward Network). The FFN contains two linear transformation layers and the activation function (ReLU) in between. The detailed equation of the FFN is as follows:

(12) $$FFN\left(x\right)=max\left(0,xW_{1}+b_{1}\right)W_{2}+b_{2},$$

where $W_{1}$ and $W_{2}$ are the weight matrices of each layer, $b_{1}$ and $b_{2}$ are their corresponding bias, and *x* is the input matrix. Finally, the matrix FFN(x) is reshaped into a one-dimensional vector $V\in R^{1,J\times S}$, which is the input to the fully connected network classifier.

### 3.6. Loss Function

The smart Ponzi scheme detection problem studied in this paper can be considered as a binary classification task. Therefore, there are two types of misclassifications:

- A non-Ponzi scheme contract is wrongly predicted to be a Ponzi scheme contract. At this point, we can manually check the smart contract to confirm security. Even if a transaction is generated, it will not cause financial loss.
- A Ponzi scheme contract is wrongly predicted to be a non-Ponzi scheme contract. Therefore, the Ponzi scheme contract will be deployed and reside on the blockchain platforms.

As the failure to find a Ponzi scheme contract can lead to large economic loss, misclassifying a Ponzi scheme contract results in a higher cost than misclassifying a non-Ponzi scheme contract. In addition, there are far fewer contracts with Ponzi schemes than the non-Ponzi scheme contracts in the smart Ponzi scheme detection dataset. The smart Ponzi scheme detection model trained on the imbalanced dataset will focus more on the non-Ponzi scheme contracts and is prone to predict that the new contract to be a non-Ponzi scheme contract. Therefore, a cost-sensitive loss function is used for the fitting. In the training set, we suppose the number of contracts with a Ponzi scheme is *u*, and the number of contracts without a Ponzi scheme is *v*. The cross entropy loss function with weights is defined as follows:

(13) $$Weight_{c}=\left\{\begin{array}{cc} p*\frac{v}{u+v} & \mathrm{if}\;c\;\mathrm{is}\;a\;\mathrm{Ponzi}\;\mathrm{scheme}\;\mathrm{contract}\\ \frac{1}{p}*\frac{u}{u+v} & \mathrm{otherwise} \end{array}\right.$$

(14) $$Loss\left(\hat{Y},Label\right)=-\sum_{c}Weight_{c}Label_{c}log\left({\hat{Y}}_{c}\right),$$

where Label is the true label, $\hat{Y}$ is the predicted outcome, and *c* denotes each contract.

### 3.7. Time Complexity Analysis

The time complexity has an important impact on the model, which qualitatively describes the running time of the algorithm. The MTCformer model proposed in this paper has the structure of both the multi-channel TextCNN and Transformer. Its specific time complexity is analyzed as follows.

There are *m* filters in the multi-channel TextCNN, where each filter has *s* convolution kernels. Furthermore, we perform *n* convolution kernel operations, and each convolution kernel size is k×d. Thus, the total time complexity of the multi-channel TextCNN is O(msnkd).

The Transformer consists of the self-attention stage and the multi-head attention stage. Let the size of *K*, *Q*, and *V* be n×d. In the self-attention stage, the time complexity of the similarity calculation, the softmax calculating for each line, and the weighted sum are $O(n^{2}d)$, $O(n^{2})$, and $O(n^{2}d)$, respectively. Therefore, the time complexity of the self-attention stage is $O(n^{2}d)$.

In the multi-head attention stage, the time complexity of the input linear projection, attention operation, and output linear projection are $O(nd^{2})$, $O(n^{2}d)$, and $O(nd^{2})$, respectively. Therefore, the time complexity of the multi-head attention stage is $O(n^{2}d+nd^{2})$.

In summary, the time complexity of MTCformer is $O\left(msnkd\right)+O\left(n^{2}d+nd^{2}\right)$. After ignoring the constant term and taking the highest power, the time complexity of MTCformer is $O(n^{2}d+nd^{2})$.

## 4. Experiment

### 4.1. Datasets

In this study, we use the same Ponzi scheme contract detection dataset as Chen et al. [7]. These contracts were collected through the API provided by etherscan.io and then manually checked for whether they are Ponzi scheme contracts by Chen et al. [7]. Before constructing the model, we recheck the results and organize them. The data from the secondary confirmation results are taken as real data. Among these smart contracts, there are 200 Ponzi contracts and 3588 non-Ponzi contracts. For most commercial projects, when investors invest in projects based on Ethereum contracts, they will require the project party to provide contract audits performed by a third party, which ensures that most smart contracts in Ethereum are not Ponzi scheme contracts. Therefore, our experimental dataset is imbalanced.

### 4.2. Evaluation Metrics

Ponzi scheme contract detection has the following four typical outputs, i.e., True Positive (TP), True Negative (TN), False Positive (FP), and False Negative (FN). TP denotes the number of the Ponzi scheme contracts that are correctly identified; TN denotes the number of the contracts without Ponzi schemes that are correctly identified; FP denotes the number of the smart contracts without Ponzi schemes that are wrongly predicted as Ponzi scheme contracts; and FN denotes the number of the Ponzi scheme contracts that are wrongly predicted as the contracts without Ponzi schemes. We use precision, recall, and F-score to evaluate the performance of the model, which are introduced as follows:

(15) $$Precision=\frac{TP}{TP+FP}$$

(16) $$Recall=\frac{TP}{TP+FN}$$

(17) $$F-score=2\times \frac{Precision\times Recall}{Precision+Recall}$$

### 4.3. Methods in Comparison

To demonstrate that MTCformer outperforms the state-of-the-art Ponzi scheme detection methods, we compare the MTCformer with the method proposed by Chen et al. [7,9] and a recently proposed deep learning method (i.e., CodeBERT). Moreover, we investigate the performance of the variants of MTCformer based on the traditional model (i.e., STC, RNN, BiLSTM, and BiGRU). These methods are briefly described as follows.

**Account**: The fraudulent nature of Ponzi schemes makes them have some unique features. These features are called account features and are readily reflected in the history of the transaction. Chen et al. [7] use the address of the smart contract to find the corresponding transaction history on the Ethereum. Account features are extracted based on transaction history for Ponzi scheme contract detection, including known rate, balance, N_investment, N_payment, difference index, paid rate, and N_maxpay. The random forest algorithm is used as the classification model based on the account features.

**Opcode**: At the level of the Ethereum Virtual Machine (EVM) [58,59], the Opcode features can also reflect the logic of the smart contract. Chen et al. [7] firstly used the Ethereum native client to obtain the bytecode, then used the disassembly tool to get the Opcode, and finally extracted all the operating codes and calculated their frequency in the contract. The random forest algorithm is used as the classification model based on the Opcode features.

**Account + Opcode**: Chen et al. [7] combined the account features and Opcode features as the composited features of a smart contract. The random forest algorithm is used as the classification model based on the composited features.

**STC**: Kim et al. [39] proposed the single-channel TextCNN method for text classification tasks using Convolutional Neural Networks. All words in a sentence are turned into a set of word vectors by word embedding as the input of CNN. In the convolutional layer, features are extracted to complete the classification.

**RNN**: This is a neural network used to process sequence data [60]. The current output of a sequence is also related to the previous output. The specific expression is that the network will memorize the previous information and apply it to the computing of the current output, and the input of the hidden layer includes not only the output of the input layer but also the output of the hidden layer at the previous moment [61].

**BiLSTM**: This is a special kind of RNN used to solve the gradient disappearance and gradient explosion problems during the training of long sequences by introducing the forget gate, input gate, and output gate [62]. The idea of Bidirectional LSTMs (BiLSTMs) is to duplicate the first recurrent layer in the network and then provide the input sequence to the first layer and a reversed copy of the input to the second, so that all available information in the past and future of a specific processing step can be considered during training [63].

**BiGRU**: This is an improved version of RNN, which is mainly improved from the following two aspects [64]. First, the influence of words at different positions in the sequence on the current hidden layer state is weighted by distance. The further the distance, the smaller the weight. Second, when an error arises, the error may be triggered by one or a few words, so only the corresponding word weight should be updated. The idea of bi-directional GRU (BiLGRU) is to include a reverse GRU in the network and then provide the input sequence simultaneously with the forward GRU so that all available information from the past and future of a particular processing step can be considered during the training process.

**CodeBERT + SVM**: Feng et al. [52] proposed a state-of-the-art pre-trained model called CodeBERT, which is built based on Transformer neural architecture and supports both natural language text and programming language as input. We use CodeBERT to extract semantic features and then employ SVM as the classifier to complete the classification. The reason we choose SVM as the classifier is that SVM performs the best among some widely used classifiers (i.e., naive Bayes, decision tree, random forest, k-nearest neighbor) according to our preliminary experimental results.

### 4.4. Parameter Setting and Experimental Procedure

In the word embedding part, we use the random initialization to generate the word embedding matrix as the input of the model. The dimension of the word embedding is 200. In the TextCNN part of the model, we choose five convolutional kernels of different sizes, which are [3,4,5,6,7] in height and 200 in width. Each convolution operation corresponds to 200 output channels. Thus, regardless of the length of the input sequence, TextCNN will always output a feature-mapping matrix of the fixed size. In the Transformer part, we set the number of hidden layer units to 200, the number of heads in the multi-head attention mechanism to 20, and the number of sub-encoder layers in the encoder to 7. The learning rate is initialized to 1 × 10${}^{-5}$. Due to the imbalance problem of the Ponzi scheme contract detection dataset, we introduce the cost-sensitive cross-entropy loss as the loss function and set the weight factor *p* to 0.8. The batch size is set to 4, and the number of epoch is 100. We choose the ReLU function as the activation function of the middle layer, the sigmoid function as the activation function of the last layer output, and AdamW [65] as the optimizer of the model.

We employ five-fold cross validation in the experiment. Specifically, we evenly divide the dataset into 5-folds. Then, we train all above-mentioned Ponzi scheme contract detection methods on 4-folds, and the left fold is used as the testing data. The procedure is repeated 5 times to result in each fold being used as training and testing data. Finally, we repeat the five-fold cross validation 10 times to reduce the variance and bias. Therefore, we can obtain 50 = (5 × 10) testing results and list the average value of the 50 results in the following tables, in which the highest value is marked in bold in each row.

### 4.5. Experimental Results

#### 4.5.1. Comparison between MTCformer and the Baselines

In order to verify the advantage of the deep learning techniques for Ponzi scheme contract detection, we first compare the MTCformer with the traditional feature-based Ponzi scheme contract detection methods, i.e., Account, Opcode, and Account + Opcode, as introduced in Section 4.3. Table 1 presents the precision, recall, and F-score values of the methods. We have the following findings in Table 1.

(1) The MTCformer performs the best in terms of all evaluation metrics, which indicates the superiority of the deep learning techniques. Specifically, it achieves a precision value of 0.97, a recall value of 0.83, and an F-score value of 0.89. The MTCformer outperforms Account by 51.56% in terms of precision, 315% in terms of recall, and 197% in terms of F-score. The MTCformer performs better than Opcode by 3.19% in terms of precision, 13.7% in terms of recall, and 8.54% in terms of F-score. The MTCformer also outperforms Account + Opcode by 2.1%, 20.29%, and 12.66% in terms of precision, recall, and F-score, respectively. To summarize, the experimental results show that the MTCformer has a powerful ability to automatically learn structural and semantic features from the smart contract code.

(2) When the random forest model is trained based on only the Account features, it achieves the lowest performance in terms of all evaluation metrics. The low recall value (0.20) indicates that the Ponzi scheme contract detection model based on the Account features is almost useless. When using the Opcode features to train the random forest model, the performance of the model is high. When the Account features and Opcode features are combined to train the random forest model, the precision of the model is further improved, but the recall and F-score values decrease.

In summary, the MTCformer achieves the highest precision, recall, and F-score values compared with the three state-of-the-art methods.

#### 4.5.2. Comparison between MTCformer and Its Variants

Table 2 presents the precision, recall, and F-score values of different variant models. We have the following findings.

(1) The single-channel TextCNN (STC) achieves a precision value of 0.94, a recall value of 0.44, and an F-score value of 0.60, respectively. The low recall value shows that the single-channel TextCNN model can only capture a part of the features of the Ponzi scheme contract. In other words, due to the limitation of a single channel, it cannot capture more complete features. By replacing the single-channel TextCNN with a multi-channel TextCNN (MTC), each filter has multiple channels (convolutional kernels), which are used to learn features that complement each other from the same filter window. When using the multi-channel TextCNN, the recall value is improved by 65.91%, and the F-score value is improved by 66%. This demonstrates that the multi-channel TextCNN has a significant improvement in capturing local features in comparison to the single-channel TextCNN. However, the recall value of the multi-channel TextCNN is only 0.73, which indicates that there are still many Ponzi scheme contracts that cannot be recognized. The potential reason is that the long-range dependencies between code tokens cannot be captured.

(2) Introducing the RNN does not improve the model performance. The reason is that RNN can only remember the nearest code tokens. However, when the distance increases, RNN cannot capture the long-range dependencies between code tokens well. However, there is an overall performance improvement after learning long-range dependencies between code tokens by adding BiLSTM, BiGRU, and Transformer. Although introducing the BiLSTM achieves the highest precision value (0.97), we hope that the Ponzi scheme contract detection model can find as many Ponzi scheme contracts as possible. Therefore, the higher recall value is more important. The Transformer has the best learning effect for long-range dependencies in terms of recall and F-score. Introducing Transformer improves the precision value by 3.2%, the recall value by 13.7%, and the F-score value by 8.5% in comparison to the multi-channel TextCNN.

(3) The CodeBERT + SVM achieves a precision value of 0.72, a recall value of 0.83, and an F-score value of 0.77, respectively. Although both CodeBERT + SVM and MTCformer achieve the same recall value, the low precision value (0.72) of CodeBERT + SVM indicates that it has high false alarms. The potential reason is that CodeBERT is pre-trained on other programming languages instead of Solidity.

In summary, the MTCformer outperforms its variants in terms of recall and F-score.

#### 4.5.3. Effectiveness of the SBT Sequence

During the processing of natural language, in addition to the semantic information of the context, structural information can also provide assistance in understanding the text. In order to verify whether structured SBT sequences highlight feature information better than unstructured code sequences, we compare the performance of the MTCformer and its variants using the SBT code sequences and plain source code as inputs. Table 3 presents the precision, recall, and F-score values of the MTCformer and its variants using the SBT code sequences and plain source code as inputs, where ’(SBT)’ represents using the SBT code sequences, and ’(PSC)’ represents using the plain source code.

The experimental results indicate that the performance of all models, except MTC and MTC + RNN, is improved. Comparing the results in Table 3, we observe that for MTC + BiLSTM, MTC + BiGRU and MTCformer, there are 1.2%, 3.6%, and 1.1% performance improvements in terms of F-score when using the structured SBT sequences as inputs. In contrast, using the structured SBT sequences is not effective for MTC and MTC + RNN. The reason is that converting the original code to SBT sequences results in a substantial increase in sequence length. Thus, there is no performance improvement for MTC and MTC + RNN, which are not good at capturing the long-range dependencies between code tokens. For models that are skilled at learning long dependencies (e.g., LSTM, GRU, and Transformer), using SBT sequences as input can yield better performance results. Therefore, using the structured code sequences generated by the SBT method can improve the overall performance of the MTCformer.

#### 4.5.4. Effectiveness of the Cost-Sensitive Loss Function

Since the smart Ponzi scheme detection dataset contains more non-Ponzi scheme contracts (i.e., the majority class samples) than Ponzi scheme ones (i.e., the minority class samples), the prediction model trained on the imbalanced dataset is prone to predict that the new contract contains a Ponzi scheme. Since misclassifying an actual Ponzi scheme contract will result in considerable economic losses, we introduce a weighted cross-entropy loss function, and a cost-sensitive parameter *p* is added to the function. Different *p* values will affect the classification performance of the model. In order to explore the effect of the parameter *p*, we set *p* to 0.7, 0.8, 0.9, 1.0, 1.1, and 1.2, respectively. In addition, the rest of the parameters are the same, and we use the SBT sequence as input. Table 4 presents the precision, recall, and F-score values of the MTCformer with different *p* values, where “-” indicates that the original cross-entropy loss function is used.

We can find that the MTCformer achieves the highest recall and F-score values when the *p* is equal to 0.8 and performs the best in terms of precision when the the *p* is equal to 1.1. Although the precision value is 1 with *p* = 1.1, the MTCformer achieves the lowest recall value (0.76), which indicates that fewer Ponzi scheme contracts can be found. We set *p* to 0.8 as the optimal parameter, since the MTCformer achieves the best overall performance. When the original cross-entropy loss function is used, the precision, recall, and F-score values are 0.94, 0.76, and 0.84, respectively. The recall value (0.76) and F-score value (0.84) are lower than those of the MTCformer using the cost-sensitive loss function with different *p* values. Therefore, introducing the cost-sensitive parameter indeed improves the performance of the MTCformer.

### 4.6. Efficiency of Different Methods

In this subsection, we focus on the efficiency of different methods. Four GeForce RTX 2080 Ti GPUs are used to train our model, and we list the training time and testing time of the eight methods in Table 5. We observe that the MTCformer requires 84.09s to train a model and 0.038s to test it. In addition, the training times of STC, MTC, MTC + RNN, MTC + BiLSTM, MTC + BiGRU, and CodeBERT + SVM are 49.71 s, 77.41 s, 81.65 s, 81.92 s, 82.10 s, and 103.98 s, respectively. The longer training time of MTCformer than STC, MTC, MTC + RNN, MTC + BiLSTM, and MTC + BiGRU is due to the multi-head attention mechanism. Although, the training time of the MTCformer is a little long, the testing time for detecting whether a smart contract is a Ponzi scheme contract is less than 0.1 s, so we argue that it is still acceptable.

## 5. Discussion

In the MTCformer, the parameters (i.e., Encoder Layer and Word Embedding Dimension) have a significant impact on the feature learning and feature representation. Therefore, this section conducts an experimental comparison to explore the best parameter combination. Furthermore, the effect of different encoder layers and word embedding dimensions of the MTCformer is discussed. We use SBT sequences as input and employ the cost-sensitive loss function with parameter p=0.8, and the remaining parameters are unchanged in the experiment. In addition, we discuss the practical implications of our study.

### 5.1. Encoder Layer

Generally, when fewer encoder layers are used, the data are poorly fitted, thus resulting in poor performance of the model. On the contrary, when more encoder layers are used, not only will the efficiency of the model training be poor, but the phenomenon of overfitting will also be triggered. In this experiment, the number of the encoder layers is set from 2 to 10, and the performance results of the MTCformer are shown in Table 6.

From Table 6, we can find that the MTCformer achieves the highest F-score value (0.89) when the number of encoder layers is equal to 7; obtains the highest precision value (0.97) with Encoder Layer = 3, 7; and performs the best in terms of recall (0.83) with Encoder Layer = 4, 5, 6, 7, and 8. When the number of encoder layers is less than 7, the model is not well-fitted. When the number of encoder layers is larger than 7, the recall value of the model shows a decreasing trend, and the overfitting phenomenon occurs. Therefore, we conclude that Encoder Layer = 7 is the optimal option for the MTCformer.

### 5.2. Word Vector Dimension

The dimension of the word vector represents the number of features of words, and a higher dimension can distinguish the words from each other more accurately. However, in practice, high-dimensional word vectors not only cause a huge memory overhead but also fade the relationship between words. On the contrary, low-dimensional word vectors also suffer from the difficulty of distinguishing different words. Therefore, we choose word vectors in the range of 100, 200, 400, 600, and 1000 in the experiment. Table 7 presents the precision, recall, and F-score values of the MTCformer with different word vector dimensions. We find that the MTCformer achieves the highest recall and F-score values when the word vector dimension is equal to 200 and performs the best in terms of precision when the word vector dimension is equal to 200 and 400. When the word vector dimension increases, the overall performance (measured by F-score) of the MTCformer tends to decrease. Therefore, we conclude that the optimal word vector dimension is 200 for the MTCformer.

### 5.3. Practical Implications

Financial fraud based on blockchain and cryptocurrencies is already a very serious problem. With the development of blockchain technology, Ponzi schemes are now under the veil of smart contracts [7,9]. From a practical perspective, the method proposed in this paper, MTCformer, has high accuracy and can actually be used to detect potential smart Ponzi schemes. Therefore, the financial losses due to fraud can be alleviated, and the transaction environment in the blockchain can be improved.

Technically speaking, the experimental results in Section 4.5.1 show that our proposed MTCformer method outperforms the hand-crafted features-based methods (i.e., Account, Opcode, and Account + Opcode) by a substantial margin. The MTCformer differs from the existing Ponzi scheme detection methods in that we employ the deep learning technique (i.e., multi-channel TextCNN and Transformer) to automatically generate discriminative features from smart contract code instead of manually designing features, which can extract the structural and semantic information of source code and result in more accurate Ponzi scheme prediction. Therefore, we suggest that subsequent researchers also take advantage of deep learning techniques to automatically extract structural and semantic features from smart contract code for better prediction.

The experimental results in Section 4.5.2 and Section 4.5.3 show that introducing the Transformer and SBT can improve the performance of MTCformer. Indeed, some researchers have employed Transformer and SBT for smart contract code analysis. For example, Yang et al. [5] proposed a smart contract code summarization approach, which leverages the SBT method to represent global semantic information of source code and Transformer to capture the long-range dependencies between code tokens. Therefore, we suggest that subsequent researchers also use the SBT method to preserve the code structure, and employ Transformer to extract the long-range dependencies for more accurate prediction in the field of smart contract code analysis.

## 6. Conclusions and Future Work

In the blockchain era, many Ponzi schemes are disguised as smart contracts, which has a significantly negative effect on the development of blockchain. The existing Ponzi scheme contract detection studies mainly focus on designing manually extracted features, which are used to train a classification model to detect Ponzi scheme contracts. However, the manually extracted features generally fail to capture the structural and semantic information of smart contract code. Therefore, in this paper, we propose a deep-learning-based Ponzi scheme detection method (i.e., MTCformer). The MTCformer firstly uses the SBT method to generate the code token sequence to retain the structural information and then utilizes the multi-channel TextCNN and Transformer to automatically extract the structural and semantic features from source code and to learn the long-range dependencies between code tokens. Our experimental results show that the MTCformer performs better than the state-of-the-art methods and their variants. In future work, we will collect more Ponzi scheme contracts to validate the generality of the MTCformer. In addition, we plan to apply our MTCformer method to other classification tasks in the field of blockchain.

## Figures and Tables

**Table 1:** The performance of different Ponzi scheme contract detection methods.

Method	*Precision*	*Recall*	*F-Score*
Account	0.64	0.20	0.30
Opcode	0.94	0.73	0.82
Account + Opcode	0.95	0.69	0.79
MTCformer	**0.97**	**0.83**	**0.89**

**Table 2:** The performance of different variant models.

Method	*Precision*	*Recall*	*F-Score*
STC	0.94	0.44	0.60
MTC	0.94	0.73	0.82
MTC + RNN	0.94	0.73	0.82
MTC + BiLSTM	**0.97**	0.76	0.85
MTC + BiGRU	0.92	0.80	0.86
CodeBERT + SVM	0.72	**0.83**	0.77
MTCformer	**0.97**	**0.83**	**0.89**

**Table 3:** The performance comparison when using the SBT sequence and plain source code as input.

Method	*Precision*	*Recall*	*F-Score*
MTC (PSC)	0.94	0.73	0.82
MTC (SBT)	0.94	0.73	0.82
MTC + RNN (PSC)	0.94	0.73	0.82
MTC + RNN (SBT)	0.94	0.73	0.82
MTC + BiLSTM (PSC)	0.94	0.77	0.84
MTC + BiLSTM (SBT)	**0.97**	0.76	0.85
MTC + BiGRU (PSC)	0.89	0.78	0.83
MTC + BiGRU (SBT)	0.92	0.80	0.86
MTCformer (PSC)	0.94	**0.83**	0.88
MTCformer (SBT)	**0.97**	**0.83**	**0.89**

**Table 4:** The performance of the MTCformer with different *p* values.

Method	*Precision*	*Recall*	*F-Score*
-	0.94	0.76	0.84
0.7	0.97	0.80	0.88
0.8	0.97	**0.83**	**0.89**
0.9	0.97	0.78	0.86
1.0	0.94	0.83	0.88
1.1	**1.00**	0.76	0.86
1.2	0.90	0.80	0.85

**Table 5:** The efficiency of different methods.

Method	*Train Time*	*Test Time*
STC	49.71 s	0.036 s
MTC	77.41 s	0.036 s
MTC + RNN	81.65 s	0.039 s
MTC + BiLSTM	81.92 s	0.065 s
MTC + BiGRU	82.10 s	0.039 s
CodeBERT + SVM	103.98 s	0.051 s
MTCformer	84.09 s	0.038 s

**Table 6:** The performance of the MTCformer with different encoder layers.

Encoder Layer	*Precision*	*Recall*	*F-Score*
2	0.94	0.78	0.85
3	**0.97**	0.80	0.88
4	0.94	**0.83**	0.88
5	0.94	**0.83**	0.88
6	0.94	**0.83**	0.88
7	**0.97**	**0.83**	**0.89**
8	0.94	**0.83**	0.88
9	0.91	0.78	0.84
10	0.91	0.76	0.83

**Table 7:** The performance of the MTCformer with different word vector dimensions.

Method	*Precision*	*Recall*	*F-Score*
100	0.94	0.83	0.88
200	**0.97**	**0.83**	**0.89**
400	**0.97**	0.80	0.88
600	0.94	0.76	0.84
1000	0.86	0.76	0.81

## Data Availability

No applicable.

## Abbreviations

AST	Abstract Syntax Tree
BiGRU	Bi-directional Gate Recurrent Unit
BiLSTM	Bi-directional Long Short-Term Memory
BIoT	Blockchain–Internet of Things
CNN	Convolutional Neural Networks
DL	Deep Learning
EOA	Externally Owned Account
EVM	Ethereum Virtual Machine
FFN	Feed Forward Network
GRU	Gate Recurrent Unit
IoT	Internet of Things
LSTM	Long Short-Term Memory
MTC	Multi-Channel TextCNN
Opcode	Operating code
PSC	Plain Source Code
RNN	Recurrent Neural Networks
SBT	Structure-Based Traversal
STC	Single-Channel TextCNN
SVM	Support Vector Machine

## References

[1] Tsankov P., Dan A., Drachsler-Cohen D., Gervais A., Buenzli F., Vechev M. Securify: Practical security analysis of smart contracts. Proceedings of the 2018 ACM SIGSAC Conference on Computer and Communications Security.

[2] Lima J.A.P., Vergilio S.R. (2020). Test Case Prioritization in Continuous Integration environments: A systematic mapping study. Inf. Softw. Technol..

[3] Röscheisen M., Baldonado M., Chang K., Gravano L., Ketchpel S., Paepcke A. (1998). The Stanford InfoBus and its service layers: Augmenting the Internet with higher-level information management protocols. Digital Libraries in Computer Science: The MeDoc Approach.

[4] Savelyev A. (2017). Contract law 2.0: ‘Smart’contracts as the beginning of the end of classic contract law. Inf. Commun. Technol. Law.

[5] Yang Z., Keung J., Yu X., Gu X., Wei Z., Ma X., Zhang M. A Multi-Modal Transformer-based Code Summarization Approach for Smart Contracts. Proceedings of the 29th IEEE/ACM International Conference on Program Comprehension (ICPC 2021).

[6] Tapscott D., Tapscott A. (2016). Blockchain Revolution: How the Technology Behind Bitcoin is Changing Money, Business, and the World.

[7] Chen W., Zheng Z., Ngai E.C.H., Zheng P., Zhou Y. (2019). Exploiting blockchain data to detect smart ponzi schemes on ethereum. IEEE Access.

[8] Vasek M., Moore T. Analyzing the Bitcoin Ponzi scheme ecosyste. Proceedings of the International Conference on Financial Cryptography and Data Security.

[9] Chen W., Zheng Z., Cui J., Ngai E., Zheng P., Zhou Y. Detecting ponzi schemes on ethereum: Towards healthier blockchain technology. Proceedings of the 2018 World Wide Web Conference.

[10] Hochreiter S., Schmidhuber J. (1997). Long short-term memory. Neural Comput..

[11] Chung J., Gulcehre C., Cho K., Bengio Y. (2014). Empirical evaluation of gated recurrent neural networks on sequence modeling. arXiv.

[12] Hu X., Li G., Xia X., Lo D., Jin Z. Deep code comment generation. Proceedings of the 2018 IEEE/ACM 26th International Conference on Program Comprehension (ICPC).

[13] Zheng X.R., Lu Y. (2021). Blockchain technology–recent research and future trend. Enterp. Inf. Syst..

[14] Singh S.K., Rathore S., Park J.H. (2020). Blockiotintelligence: A blockchain-enabled intelligent IoT architecture with artificial intelligence. Future Gener. Comput. Syst..

[15] Tsang Y., Wu C., Ip W., Shiau W.L. (2021). Exploring the intellectual cores of the blockchain–Internet of Things (BIoT). J. Enterp. Inf. Manag..

[16] Zhang Y., Wen J. (2017). The IoT electric business model: Using blockchain technology for the internet of things. Peer-to-Peer Netw. Appl..

[17] Puri V., Priyadarshini I., Kumar R., Van Le C. (2021). Smart contract based policies for the Internet of Things. Clust. Comput..

[18] Zhang Y., Kasahara S., Shen Y., Jiang X., Wan J. (2018). Smart contract-based access control for the internet of things. IEEE Internet Things J..

[19] Ellul J., Pace G.J. Alkylvm: A virtual machine for smart contract blockchain connected internet of things. Proceedings of the 2018 9th IFIP International Conference on New Technologies, Mobility and Security (NTMS).

[20] Buterin V. (2014). A next-generation smart contract and decentralized application platform. White Pap..

[21] Song J.G., Kang E.S., Shin H.W., Jang J.W. (2021). A Smart Contract-Based P2P Energy Trading System with Dynamic Pricing on Ethereum Blockchain. Sensors.

[22] Wang S., Ouyang L., Yuan Y., Ni X., Han X., Wang F.Y. (2019). Blockchain-enabled smart contracts: Architecture, applications, and future trends. IEEE Trans. Syst. Man Cybern. Syst..

[23] Song J.G., Moon S.J., Jang J.W. (2021). A Scalable Implementation of Anonymous Voting over Ethereum Blockchain. Sensors.

[24] Bian L., Zhang L., Zhao K., Wang H., Gong S. (2021). Image-Based Scam Detection Method Using an Attention Capsule Network. IEEE Access.

[25] Ngai E.W., Hu Y., Wong Y.H., Chen Y., Sun X. (2011). The application of data mining techniques in financial fraud detection: A classification framework and an academic review of literature. Decis. Support Syst..

[26] Bartoletti M., Pes B., Serusi S. Data mining for detecting bitcoin ponzi schemes. Proceedings of the 2018 Crypto Valley Conference on Blockchain Technology (CVCBT).

[27] Shippey T., Bowes D., Hall T. (2019). Automatically identifying code features for software defect prediction: Using AST N-grams. Inf. Softw. Technol..

[28] Huang Y., Huang S., Chen H., Chen X., Zheng Z., Luo X., Jia N., Hu X., Zhou X. (2020). Towards automatically generating block comments for code snippets. Inf. Softw. Technol..

[29] Yuan W., Nguyen H.H., Jiang L., Chen Y., Zhao J., Yu H. (2019). API recommendation for event-driven Android application development. Inf. Softw. Technol..

[30] LeClair A., Jiang S., McMillan C. A neural model for generating natural language summaries of program subroutines. Proceedings of the 2019 IEEE/ACM 41st International Conference on Software Engineering (ICSE).

[31] Wei B., Li G., Xia X., Fu Z., Jin Z. (2019). Code generation as a dual task of code summarization. arXiv.

[32] Hu X., Li G., Xia X., Lo D., Jin Z. (2020). Deep code comment generation with hybrid lexical and syntactical information. Empir. Softw. Eng..

[33] Yih W.T., He X., Meek C. Semantic parsing for single-relation question answering. Proceedings of the 52nd Annual Meeting of the Association for Computational Linguistics (Volume 2: Short Papers).

[34] Xiao Y., Keung J., Bennin K.E., Mi Q. (2019). Improving bug localization with word embedding and enhanced convolutional neural networks. Inf. Softw. Technol..

[35] Zhou P., Liu J., Liu X., Yang Z., Grundy J. (2019). Is deep learning better than traditional approaches in tag recommendation for software information sites?. Inf. Softw. Technol..

[36] Jiang Y., Lu P., Su X., Wang T. (2020). LTRWES: A new framework for security bug report detection. Inf. Softw. Technol..

[37] Shen Y., He X., Gao J., Deng L., Mesnil G. Learning semantic representations using convolutional neural networks for web search. Proceedings of the 23rd International Conference on World Wide Web.

[38] Kalchbrenner N., Grefenstette E., Blunsom P. (2014). A convolutional neural network for modelling sentences. arXiv.

[39] Kim Y. (2014). Convolutional Neural Networks for Sentence Classification. arXiv.

[40] Guo B., Zhang C., Liu J., Ma X. (2019). Improving text classification with weighted word embeddings via a multi-channel TextCNN model. Neurocomputing.

[41] Conneau A., Schwenk H., Barrault L., Lecun Y. (2016). Very deep convolutional networks for text classification. arXiv.

[42] Chen T., Xu R., He Y., Wang X. (2017). Improving sentiment analysis via sentence type classification using BiLSTM-CRF and CNN. Expert Syst. Appl..

[43] Li S., Zhao Z., Liu T., Hu R., Du X. Initializing convolutional filters with semantic features for text classification. Proceedings of the 2017 Conference on Empirical Methods in Natural Language Processing.

[44] Yenigalla P., Kar S., Singh C., Nagar A., Mathur G. Addressing unseen word problem in text classification. Proceedings of the International Conference on Applications of Natural Language to Information Systems.

[45] Zhang Y., Wang Q., Li Y., Wu X. (2018). Sentiment classification based on piecewise pooling convolutional neural network. Comput. Mater. Contin..

[46] Rezaeinia S.M., Ghodsi A., Rahmani R. (2018). Text classification based on multiple block convolutional highways. arXiv.

[47] Vaswani A., Shazeer N., Parmar N., Uszkoreit J., Jones L., Gomez A.N., Kaiser L., Polosukhin I. (2017). Attention is all you need. arXiv.

[48] Tian J., Xing W., Li Z. (2020). BVDetector: A program slice-based binary code vulnerability intelligent detection system. Inf. Softw. Technol..

[49] Cai H., Fu X., Hamou-Lhadj A. (2020). A study of run-time behavioral evolution of benign versus malicious apps in android. Inf. Softw. Technol..

[50] Hussain Y., Huang Z., Zhou Y., Wang S. (2020). CodeGRU: Context-aware deep learning with gated recurrent unit for source code modeling. Inf. Softw. Technol..

[51] Parr T.J., Quong R.W. (1995). ANTLR: A predicated-LL (k) parser generator. Softw. Pract. Exp..

[52] Feng Z., Guo D., Tang D., Duan N., Feng X., Gong M., Shou L., Qin B., Liu T., Jiang D. (2020). Codebert: A pre-trained model for programming and natural languages. arXiv.

[53] Mikolov T., Chen K., Corrado G., Dean J. (2013). Efficient estimation of word representations in vector space. arXiv.

[54] Pennington J., Socher R., Manning C.D. Glove: Global vectors for word representation. Proceedings of the 2014 Conference on Empirical Methods in Natural Language Processing (EMNLP).

[55] Bojanowski P., Grave E., Joulin A., Mikolov T. (2017). Enriching word vectors with subword information. Trans. Assoc. Comput. Linguist..

[56] Peters M.E., Neumann M., Iyyer M., Gardner M., Clark C., Lee K., Zettlemoyer L. (2018). Deep contextualized word representations. arXiv.

[57] Voita E., Talbot D., Moiseev F., Sennrich R., Titov I. (2019). Analyzing multi-head self-attention: Specialized heads do the heavy lifting, the rest can be pruned. arXiv.

[58] Atzei N., Bartoletti M., Cimoli T. A survey of attacks on ethereum smart contracts (sok). Proceedings of the International Conference on Principles of Security and Trust.

[59] Chen T., Li X., Luo X., Zhang X. Under-optimized smart contracts devour your money. Proceedings of the 2017 IEEE 24th International Conference on Software Analysis, Evolution and Reengineering (SANER).

[60] Perticas C.F., Indurkhya B. (2020). Neural networks learn to detect and emulate sorting algorithms from images of their execution traces. Inf. Softw. Technol..

[61] Wang L., Wang W. Research and Construction of Junior High School Subject Q&A System Model based on Deep Learning. Proceedings of the 2018 International Conference on Information Systems and Computer Aided Education (ICISCAE).

[62] Ochodek M., Kopczyńska S., Staron M. (2020). Deep learning model for end-to-end approximation of COSMIC functional size based on use-case names. Inf. Softw. Technol..

[63] Al-Azani S., El-Alfy E.S. Emojis-based sentiment classification of Arabic microblogs using deep recurrent neural networks. Proceedings of the 2018 International Conference on Computing Sciences and Engineering (ICCSE).

[64] Zhu Z., Dai W., Hu Y., Li J. (2020). Speech Emotion recognition model based on Bi-GRU and focal loss. Pattern Recognit. Lett..

[65] Loshchilov I., Hutter F. (2017). Fixing Weight Decay Regularization in Adam. arXiv.

